# Consumers' Response to Sugar Label Formats in Packaged Foods: A Multi-Methods Study in Brazil

**DOI:** 10.3389/fnut.2022.896784

**Published:** 2022-06-16

**Authors:** Tailane Scapin, Ana Carolina Fernandes, Maria Shahid, Simone Pettigrew, Neha Khandpur, Greyce Luci Bernardo, Paula Lazzarin Uggioni, Rossana Pacheco da Costa Proença

**Affiliations:** ^1^Nutrition in Foodservice Research Centre (NUPPRE), Nutrition Postgraduate Program (PPGN), Federal University of Santa Catarina (UFSC), Florianópolis, Brazil; ^2^The George Institute for Global Health, Faculty of Medicine, University of New South Wales, Sydney, NSW, Australia; ^3^Department of Nutrition, Faculty of Public Health, University of São Paulo, São Paulo, Brazil; ^4^Department of Nutrition, Harvard T.H. Chan School of Public Health, Boston, MA, United States

**Keywords:** food labelling, sugary foods, health claims, warning labels, consumer behaviour, trial

## Abstract

Providing information about the sugar content of packaged foods on product labels is an important strategy to lower consumers' sugar intake. This study assessed the effect of exposure to different sugar labels on consumers' understanding of the sugar content of foods and their food choices. In the first phase, five focus groups were conducted with a convenience sample of Brazilian adults to explore their perceptions about food labelling in general and sugar labelling in particular. Based on the qualitative results, four sugar label formats were developed and subsequently tested in a five-arm study on 1,277 adults *via* a randomised controlled online survey. The formats were: (i) no sugar information—control, (ii) total and added sugar content displayed in the Nutrition Information Panel (NIP), (iii) a front-of-package (FoP) octagonal warning for “high-in-sugar” products, (iv) a FoP magnifying glass warning for “high-in-sugar” products, and (v) a “high-in-sugar” warning text embedded on the NIP. Participants from the focus groups reported being confused about the meaning of “sugar” and “added sugar” on food labels and indicated that more interpretive labels, such as the FoP warnings, would help them choose products with low sugar content. In the experiment, all intervention sugar label formats improved participants' understanding of the sugar content of the tested food products, with the FoP warnings (iii and iv) showing the best results. While non-significant differences among label conditions were observed for food choices, the FoP octagonal warning prompted participants to choose high-in-sugar products less often. Given current public policy agendas aiming to reduce added sugar intake, there is a need to strengthen food labelling policies and nutrition disclosure policies that target the display of added sugar and build consumer awareness in using these tools to avoid high-in-sugar products.

## Introduction

Brazil, like many countries around the world, is facing the increasing burden of non-communicable diseases (NCDs) across its population (1, 2). Up to 4% of the global disease burden has been related to an unhealthy diet (3), making diet an important modifiable behavioural risk factor for NCDs. Of the several aspects of an unhealthy diet, the excessive consumption of sugar has been associated with the development or aggravation of several NCDs (4–6). “Sugars” is the generic name of a group of monosaccharides (glucose, galactose, and fructose) and disaccharides (sucrose, lactose, maltose, and trehalose). Colloquially, the term “sugar” is usually used to refer solely to sucrose or refined sugar—also known by table sugar (7). Sugars can be classified as intrinsic, added or free, and total for dietary purposes. Intrinsic sugars are found naturally within whole fruits, vegetables, dairy, and grains. Added sugars include sugars and syrups added during the processing of foods (such as sucrose or dextrose) (8). The definition of free sugar includes added sugars and further includes sugars found naturally within fruit juices and fruit purees of all concentrations. Total sugar include all sugar types (9). Further discussion around sugar definitions can be found elsewhere (7, 10). In Brazil, 64% of the adult population is eating more free sugar than recommended by the World Health Organization (WHO) (11), making the country the world's fourth-largest consumer of sucrose (12). Table sugar and sugary packaged foods are among the main sources of free sugars intake (13).

Following the recommendations in the WHO guidelines (9), many countries are considering regulations or public health policy measures aiming at lowering sugar intake in their population. Sugar labelling is located among these actions and has been gaining prominence in health agendas worldwide as a strategy to inform consumers about the sugar content of packaged foods. Countries such as the United States, Australia, New Zealand, and members of the European Union follow the Codex Alimentarius recommendation on food labelling, which states that total sugar content should be displayed on labels (14). Requirements for declaration of added sugar are now also being implemented in some countries. The United States, for example, required the inclusion of the amount of both total and added sugars in the nutrition facts panel by 2021 (15).

In Latin America, some countries have been establishing regulations on added sugar front-of-pack (FoP) warning labels to help consumers avoid high-in-sugar products. FoP labelling includes simplified information about nutritional content or health aspects of foods, and they are displayed on the front of the package to assist consumers make healthier food choices during their quick decision-making shopping process (16). In Chile, a FoP octagonal warning label stating “high-in-sugar” is mandatory for food products exceeding defined sugar content thresholds (17), and this same format has been implemented in Peru (18) and Uruguay (19) and approved to be implemented in Mexico (20).

In Brazil, 71% of the packaged food available for sale at the supermarket has at least one type of added sugar ingredient (21). However, it is not a requirement for total or added sugar contents to be displayed on labels as the listing of this information is not mandatory under Brazil's food labelling regulations from 2003—which is still enforced (22). In 2014, through The National Health Surveillance Agency (ANVISA—Agência Nacional de Vigilância Sanitária), the Brazilian Ministry of Health began debating the Brazilian food labelling regulation, including the implementation of a sugar label format. A preliminary report from this discussion reinforced the need to declare sugar on the back-of-pack Nutrition Information Panel (NIP) and to implement a FoP warning label for high-in-sugar products. At the time, a lack of evidence prevented ANVISA from deciding which format would be most effective to help Brazilian consumers identify sugar amounts through labels and discourage the selection of high-in-sugar foods (23). At the end of 2020, the Brazilian government announced the final changes for the food labelling regulation in Brazil. These changes included the mandatory declaration of total and added sugar content in grammes in the NIP and a FoP magnifying glass warning indicating that a product is high in sugar (24). The magnifying glass format was put forth by ANVISA and it seems to be based on discussions made by the government of Canada in 2017 (25), but without extensive evidence of this format efficacy on consumers' food choices (26). Although the changes in the Brazilian food labelling rules were published in 2020, food manufacturers are not mandated to apply these changes on the label of their products until October 2022. At the present point in time, the list of ingredients declared on the packages is the only mandatory information to consumers identify if a product has added sugar ingredients in its composition.

Studies investigating consumers' understanding of food labels and their influence on food choices in the Brazilian population are sparse (27–29), and they demonstrated a preference for labels in the form of FoP warnings. Given the gap in this area of research, this study focuses on sugar and provides additional information on consumer preference for the presentation of this information by exploring Brazilian consumers' responses to different sugar label formats. Specific study objectives include to: (a) explore consumer perceptions of what “sugar” means and which label features would help them to identify sugar ingredients in packaged foods; (b) compare the effectiveness of four different sugar label formats in improving consumers' understanding of the sugar levels in a set of products; and (c) evaluate the influence of the four label formats on consumers' food choices.

## Materials and Methods

A multi-method approach encompassing qualitative and quantitative phases was used to choose and test sugar label formats for packaged food products. Initially, focus groups were conducted with a convenience sample of young adult food-label users to explore perceptions of three pre-defined sugar label formats. The results were used to adapt the formats to be tested in a survey evaluating the influence of sugar label formats on consumers' understanding of the sugar content in packaged foods and food choices. These two data collection phases are described further below.

### Qualitative Phase

#### Methods

Five focus groups were conducted in the city of Florianopolis (south Brazil) during June and July of 2019 with a convenience sample of 32 young adults (18–33 years). Young adults were chosen since they usually have a high intake of added sugar, mainly sourced from packaged food and beverages (13). Only participants who self-reported usually using food label information during their food shopping were included to ensure they had previous experience with the subject of this study. Rather than seek findings generalisation, this qualitative phase attempted to find examples of behaviours and clarify the thoughts and feelings of individuals with a previous experience of the phenomena of interest (food label use) in order to produce evidence for developing the quantitative phase of this study.

Focus group size ranged from four to nine participants, with a mean group size of six participants. The mean age was 23 years (±4.1), 50% of the sample was female, 75% were undergraduate students, and 31% had at least one dietary restriction (mainly in relation to lactose intolerance). Participants were recruited *via* posts on social media platforms for university study groups, flyers shared in the university campus, and snowballing among those registered for the study (i.e., participants who participate in the study were asked to invite relatives and friends to take part of the study). Individuals with training in nutrition were not included. Further details about the qualitative phase are available elsewhere (30).

**Figure 1:**
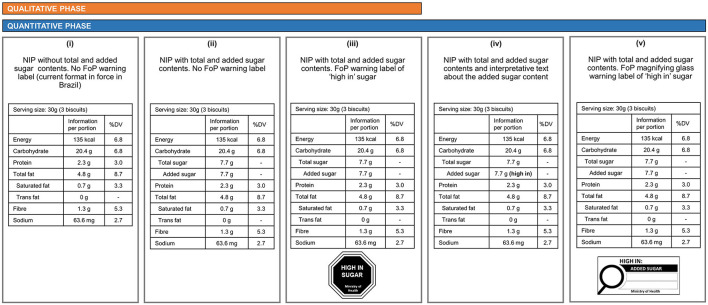
Sugar label conditions used in the qualitative and quantitative phases. The ingredient list of each product was also provided during the survey. Formats (i), (ii), and (iii) were used in the qualitative phase. All formats were used in the quantitative phase. NIP, Nutrition Information Panel; FoP, Front-of-Package; DV, Daily Value.

The first author moderated all focus groups with the support of two observers who took notes. A semi-structured interview guide was developed based on the literature, including our previous systematic review investigating sugar labelling formats and consumers' understanding (31). The interview guide included open-ended questions that covered participants' perception of sugar (e.g., What do you understand by sugars? What do you think “total sugar” and “added sugar” declared on food labels mean?), food labelling use (e.g., What do you think about food labelling information?), and reactions (e.g., How would you identify if this is a high-in-sugar biscuit? Would this format assist you while choosing a food product?) to three different formats of food labels carrying information about sugar (Figure 1). The labels were fixed on real packages of a well-known brand of biscuit sold in Brazil. The order of presentation of the sugar label formats was from the least interpretative format (i), followed by some interpretation (ii), and, finally, the most interpretive one (iii). Participants had time to hold and observe the packages before they were invited to express their perceptions regarding the label formats, including how well they understood the information in each format and how useful this information would be for their food choices. The groups lasted from 45 to 70 min and were audio-recorded. The recordings were transcribed verbatim and imported into MAXQDA software (VERBI GmbH, Pty Ltd) for thematic analysis (32).

#### Findings

Most of the participants demonstrated a low understanding of sugar and which names sugar is called on food labels. Almost all participants indicated they had never heard the term “added sugars” before, and many of them were confused when both “total sugar” and “added sugar” information was presented on food labels. Many participants were also confused about the differences between sugars and carbohydrates, and assumed that these terms were synonymous. There was a consensus among participants that food labels should provide clear and easy-to-understand information about sugar to support consumers' food choices.

Participants unanimously perceived the first format [(i) no information about sugar on the NIP] as being the least useful for food choices. Many participants mentioned that with this format, they rely only on the confusing and small-font-size list of ingredients to determine the presence of sugar in a product and that the names of some sugar ingredients were unfamiliar to them. The second format [(ii) total sugar and added sugar contents listed in the NIP] was preferred over the first format to provide information about the exact quantity of sugar in a food product and to compare products within the same food category. Although participants at first demonstrated confusion about the differences in “total sugar” and “added sugar” contents, they found the space gap included in the heading “added sugars” under the “total sugar” sugar beneficial to identify those sugars were part of the total sugar content.

*“Now I understood the logic here, this space gap [talking about the gap space in the headings for total and added sugar contents] is to show us that the added sugars are part of the total sugars and all of them are part of the carbohydrates. I always thought all carbs were sugar, but since the contents of carbohydrates and sugars are different here [format 2], I can see they are not the same thing!” (Male, focus group 1)*.

When asked to evaluate if the biscuit had a high added sugar content, most participants experienced difficulty determining what constitutes “high” content only by looking at the total and added sugar content only. Some participants suggested including some kind of interpretation such as a “high-in-sugar” text close to the amount of sugar on the NIP for better understanding of the sugar content.

*“I liked this format [format 2] because it gives me the information about the sugar content of this biscuit, but I think it would be good if they [food manufacturers] include some interpretation close to the sugar content to tell me “Caution, this is a high-in-sugar product”—that would help me as I don't know how to interpret the numbers here” (Female, focus group 4)*.

The NIP + octagonal warning label (iii) was chosen as the most useful to obtain consumers' attention and facilitate quick interpretation of a product's sugar content. Many participants believed that this format could help consumers demystify which products are high in sugar but also have a health halo (e.g., cereal bars and whole-grain biscuits). As the discussions evolved, many participants suggested that format (iii) would suit their needs for quantitative information about sugar (NIP) and a quick way to determine the sugar level of a product (warning) at the point of purchase, influencing their choice for lower sugar products. A few participants also indicated that any warnings or message on labels should be endorsed by the Health Ministry and that this endorsement should appears on labels. In addition, many participants felt that information voluntarily provided by the food industry is untrustworthy.

*“For me, this format with a warning [format 3] would be the best as it gives me the information straight away (Male, focus group 2)*.

*But if someone needs to compare two products, the best for me would also have the sugar content information on the back. Then, even if I am choosing between two high-in-sugar products, I can see which of them is lower in sugar and pick that one… or I would probably avoid both [laugh] (Female, focus group 2).”*

### Quantitative Phase

#### Label Conditions

For the quantitative phase of the study, the three label formats used in the qualitative phase [(i), (ii), and (iii)], and two more formats [(iv) and (v)] were included (Figure 1). Format (iv) was included because it was proposed by a public consultation for front-of-pack labels made by ANVISA which emerged in between the qualitative and quantitative phases of this study. This format includes a magnifying glass warning indicating when a product is high in sugar (Figure 1). Format (v) includes interpretive “high in” sugar text embedded in the NIP, which was suggested during the focus groups discussion. The two formats with FoP information [(iii) and (iv)] also had text saying “Ministry of Health of Brazil,” as suggested during the focus groups. The decision to test sugar labelling formats for both the FoP and the back of pack (NIP) labels was made to align with the changes in the Brazilian food label rules, which will include modifications in both sources of nutritional information on food packages. Three food categories (whole-grain biscuit, cereal bar, and yoghourt) were tested in the quantitative phase. They were selected because they are commonly available in Brazilian supermarkets, have brands with different sugar levels, and are often misperceived as healthy.

#### Study Sample

**Figure 2:**
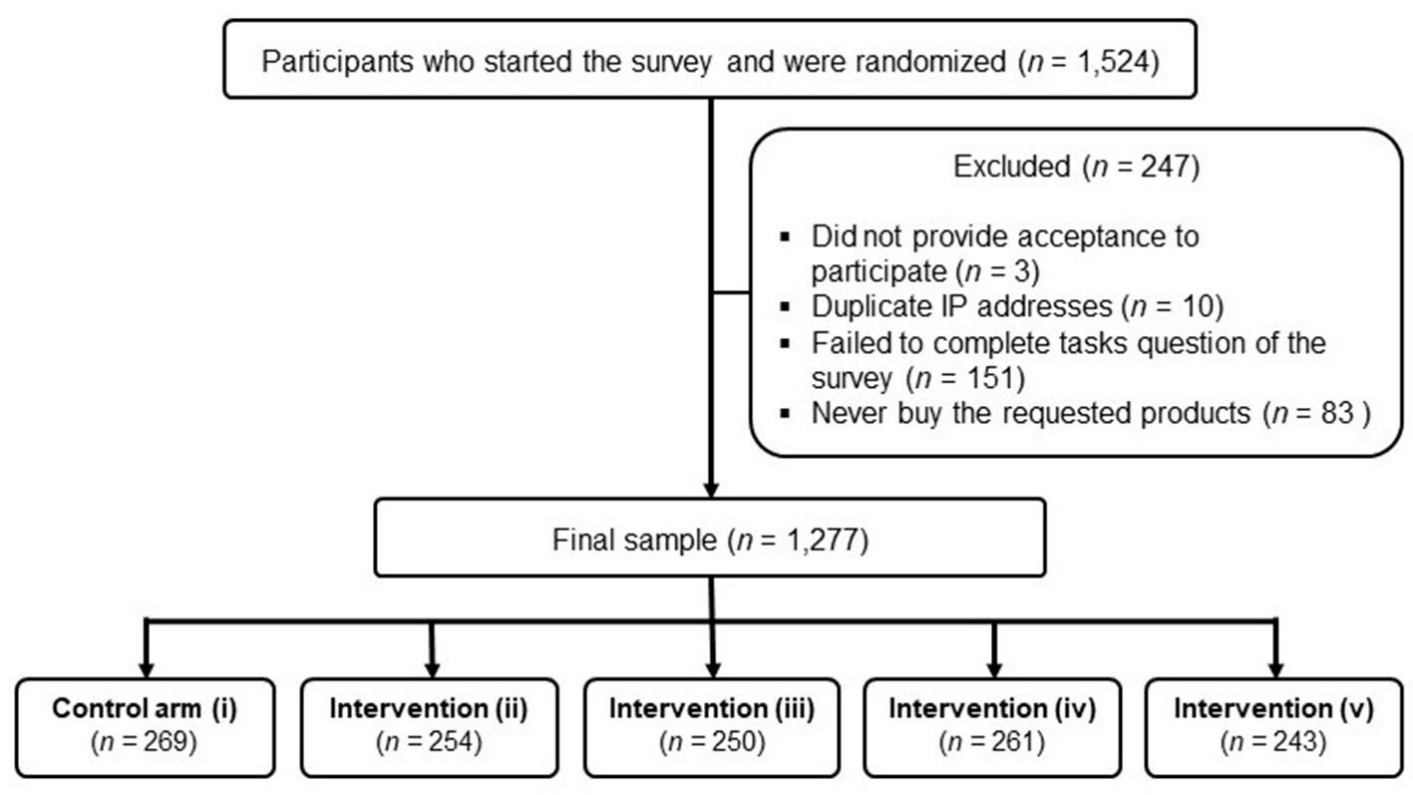
Flowchart of the participants included in the study.

An online randomised controlled experiment was conducted in Portuguese over a period of 6 weeks between May and June of 2020. Participants were recruited from posts on social media platforms of university study groups, e-mail lists of consumer association groups across Brazil, and via snowball technique. To avoid the possibility of the same person taking the survey multiple times, IP address information from the device used by the participant to take the survey was collected and duplicate IP address were removed. A virtual link providing access to the survey hosted on the Qualtrics® platform was created and shared in the ads for this study. People were eligible to participate if they were 18 years or older, provided consented to participate, and had access to a computer or tablet with internet access. At the beginning of the survey, participants were asked to provide information on sex, age, region of residence, education level, self-reported weight and height, dietary restrictions, and self-estimated level of health awareness and nutrition knowledge. They were also asked to declare the frequency of purchase of the tested food categories on a four-point scale (“Always,” “Often,” “Sometimes” and “Never”). Those who responded “Never” to all the three food categories were excluded to ensure responses reflected real-world food choice behaviours. A total of 1,524 people accessed the survey, of whom 1,277 fit the eligibility criteria and completed the survey (Figure 2).

#### Study Design and Stimuli

For each food category, a set of three food products with different levels of added sugar was created (total of nine different products). Two of the three food products were high-in-sugar options, as defined by the parameters established by the Nutrient Profile Model of the Pan-American Health Organization (33). To approximate the task with a real-world food choice scenario, food products selected for this study were well-known brands available in Brazilian supermarkets. All products under the same food category had their nutrition information standardised to the same serving size to help consumers compare the products during the tasks.

Following the sociodemographic, lifestyle and nutrition-related questions, participants were randomly allocated to a single label format and asked to complete choice and understanding tasks. The image of the product's front panel was provided on the left-hand side of the survey screen, with the respective NIP formats presented on the right-hand side. Participants allocated on FoP label formats [label conditions (iii) and (iv)] saw this information incorporated directly into the products' image of the front panel, affixed at the same place in each package, covering the same area on the packages. The list of ingredients of all foods was also provided on the screen for the five label conditions. Participants had the option to zoom in on the images of the food products during the tasks. Any other nutritional information or quality indicators (e.g., nutrition claims) were digitally removed from the packages to avoid unduly influencing participants' perceptions of the food products. Examples of the stimuli used in the survey are displayed in Supplementary Figure S1.

#### Study Procedures

Participants saw the same food products and responded to the same set of questions for all the label conditions. The presence and type of label format on the food products were the only aspects that differed across arms. To minimise priming participants to pay attention to the label formats and modify their choices accordingly, food choice was measured first for each product category, and then participants answer questions about sugar content of the presented products.

First, participants viewed the three sets of food products one at a time. For each set they were asked to select which of the three displayed products they would choose to purchase, with an “I would not choose any of these products” option also available. Participants who selected at least one product in this task were requested to indicate the main reason for their choice for each food category across the following options that had their order randomised: “Brand preference,” “Nutritional information,” and “Taste.” In the second part of the survey, participants were presented with the same set of food products and asked to nominate which of the three products had the highest amount of sugar. All choice tasks were completed for all food categories first, followed by the understanding tasks for all food categories. The order of presentation of the food categories was randomised between respondents. Finally, the food label condition to which the participant has been assigned was presented as a medium-sized image in the middle of the screen and participants were asked “Did you notice this information on the label on the previous questions?” with a yes/no answer option. if they noticed the label while completing the survey. They were then requested to indicate on a 7-point Likert scale (1—totally disagree/7—totally agree) whether they agreed or disagreed with these statements: “This label influenced my food choices in this survey” and “This label makes it easy to understand the amount of sugar in the food product”.

#### Statistical Analysis

Chi-squared and one-way ANOVA tests were conducted to test for differences in sociodemographic (age, sex, the region of living, and education) and health variables (BMI, health awareness, and nutrition knowledge) between label conditions at a 0.05 alpha level. For the understanding outcome, the proportions of participants who correctly nominated the product with the highest sugar content were calculated by food category and summarised for all categories (a maximum of three possible correct answers). For the food choice outcome, the proportions of participants who selected a high-in-sugar product were calculated for each product category and across all three food categories. For the questions “This label influenced my food choices in this survey” and “This label makes it easy to understand the amount of sugar in the food product,” data were presented as the proportion of agreement by summarising the “Strongly agree,” “Agree,” and “Somewhat agree” points from the 7-points Likert scale.

Chi-squared tests were used to evaluate the relationship between label conditions and the understanding and food choice outcomes, with significance set at a Bonferroni-corrected alpha level of 0.01 [α/*n* = 0.05/5] as suggested in the literature (34). Data were treated as dichotomous variables for the understanding outcome (selecting the correct vs. the wrong answers) and the food choice outcome (selecting a high-in-sugar product vs. a non-high-in-sugar product). Participants who selected the option “I wouldn't choose any of these products” during the food choice task had results presented separately and were removed from the association analysis for the related food category as they have not chosen any product. Sensitivity analyses were performed following the exclusion of participants who did not recall seeing the label intervention during the survey. A binary logistic regression model was used to test for differences in odds ratios for the understanding outcome by label condition. All analyses were conducted using STATA/IC software version 13.0 (College Station, TX: StataCorp. 2009).

### Ethics

Both phases of this research were approved by the Ethics Committee of the University where the study was conducted (No. 3063750) and performed in accordance with the ethical standards laid out in the Declaration of Helsinki. All participants were volunteers and provided informed consent before completing the study.

## Results

### Participants' Characteristics

Sociodemographic, lifestyle and nutrition-related characteristics of the study population are presented in Table 1. The final sample included 1,277 Brazilian participants, of whom 78% were women, 72% were enrolled in or had an undergraduate degree, and the mean age was 33.0 (±11.7) years. Participants from all regions of the country were surveyed, with most living in the South and Southeast regions (79%). Most participants (84%) reported using food label information “always” or “often,” and they presented a high median (5.7 for a 7-points Likert scale) of self-reported health awareness and nutrition knowledge. No significant differences in sociodemographic and health variables between label conditions were found.

**Table 1:** Participant characteristics, total and by label condition.

**Characteristics**	**Total sample** **(*n* = 1,277)**	**i. Control group** **(*n* = 269)**	**ii. Proposed** **NIP (*n* = 254)**	**iii. Proposed** **NIP + FoP octagonal** **warning** **(*n* = 250)**	**iv. Proposed** **NIP + FoP magnifying glass warning (*n* = 261)**	**v. Proposed** **NIP + “high in sugar” text** **(*n* = 243)**	***p*-value**
Age mean years (SD)	33.0 (±12.7)	32.3 (±11.2)	32.8 (±11.8)	33.4 (±12.0)	33.9 (±12.5)	32.8 (±11.1)	0.560
**Sex**, ***n*** **(%)**							
Female	1,001 (78%)	211 (78%)	199 (78%)	194 (78%)	204 (78%)	193 (79%)	0.993
Male	276 (22%)	58 (22%)	55 (22%)	56 (22%)	57 (22%)	50 (21%)	
**Education** ***n*** **(%)**							
High school or less	353 (28%)	76 (28%)	75 (30%)	67 (27%)	71 (27%)	64 (26%)	0.676
Undergraduate	309 (24%)	70 (26%)	60 (24%)	54 (22%)	67 (26%)	58 (24%)	
MBA	219 (17%)	38 (14%)	43 (17%)	39 (16%)	53 (20%)	46 (19%)	
Master/PhD	396 (31%)	85 (32%)	76 (30 %)	90 (36%)	70 (27%)	75 (31%)	
BMI mean kg/square metre (SD)	24.2 (±4.3)	24.1 (±4.1)	24.3 (±4.3)	23.8 (±4.6)	24.6 (±4.5)	24.2 (±4.2)	0.252
**Region of the country** ***n*** **(%)**							
North/Northeast	171 (13%)	31 (12%)	33 (13%)	35 (14%)	36 (14%)	36 (15%)	0.925
Central-west	95 (7%)	16 (6%)	19 (8%)	21 (8%)	19 (7%)	20 (8%)	
South/Southeast	1,011 (79%)	222 (83%)	202 (80%)	194 (78%)	206 (79%)	187 (77%)	
**Dietary restriction** ***n*** **(%)**							
Yes	287 (23%)	60 (22%)	58 (23%)	57 (23%)	51 (20%)	61 (25%)	0.682
**Frequency of nutrition label use**^**a**^ ***n*** **(%)**							
Always/often	1,057 (84%)	222 (85%)	199 (80%)	217 (87%)	223 (86%)	196 (81%)	0.107
Sometime/never	200 (15%)	38 (15%)	49 (20%)	32 (13%)	35 (14%)	46 (19%)	
Health awareness^b^, mean (SD)	6.1 (±1.0)	6.0 (±1.0)	6.1 (±0.9)	6.1 (±1.0)	6.1 (±1.1)	6.0 (±1.0)	0.571
Nutrition knowledge^c^, mean (SD)	5.7 (±1.4)	5.7 (±1.3)	5.6 (±1.5)	5.7 (±1.3)	5.6 (±1.3)	5.6 (±1.4)	0.543
Noticed the label in the survey, *n* (%)^a^	992 (79%)	227 (87%)^iii, iv^	218 (88%)^iii, iv^	166 (65%)^i, ii, v^	166 (64%)^i, ii, v^	215 (89%)^iii, iv^	<0.001

*Number superscripts (e.g.,^i, ii, iii^)indicate that a result is significantly different from the study condition with the corresponding number based on Bonferroni-corrected post hoc tests with an alpha set at 0.01.*

a *Different sample size for this question (n = 1,257)*.

b *Measured by the question “I reflect a lot about my health” on a 7-point Likert scale, 1= strongly disagree, 7= strongly agree*.

c *Measured by the question “I know a lot about Nutrition” on a 7-point Likert scale, 1= strongly disagree, 7= strongly agree. NIP, Nutrition Information Panel; FoP, Front-of-Package*.

### Understanding (Primary Outcome)

The proportions of correct answers for each label condition and type of food are presented in Table 2. Compared to the control condition, the proportion of participants who correctly selected the product with the highest sugar content was significantly higher in all intervention groups, except in the case of the yoghourt category. Comparisons across the intervention formats showed no significant differences in the proportion of correct answers. In the sensitivity analyses, which included only participants who recalled seeing the label formats tested during the survey, results remained similar for whole-grain biscuit, yoghourt, and overall. For the cereal bar category, only interventions (iv) [proposed NIP plus magnifying glass warning] and (v) [proposed NIP plus high in sugar text] were significantly different from the control condition (Supplementary Table S1).

**Table 2:** Participants' understanding of the sugar content of the products and their perceived understanding of the labels [*n* (%)], by study arm (*n* = 1,277).

**Outcomes**	**Total sample** **(*n* = 1,277)**	**i. Control group** **(*n* = 269)**	**ii. Proposed NIP (*n* = 254)**	**iii. Proposed NIP + FoP octagonal warning** **(*n* = 250)**	**iv. Proposed NIP + FoP magnifying glass warning (*n* = 261)**	**v. Proposed NIP + ‘high in sugar’ text** **(*n* = 243)**	***p*-value**
**Understanding**							
The proportion of correct answers about which product had the highest sugar content
Whole-grain biscuits	1,125 (88)	208 (77)^ii, iii, iv, v^	234 (92)^i^	232 (93)^i^	234 (90)^i^	217 (89)^i^	<0.001
Cereal bars	1,204 (94)	234 (87)^ii, iii, iv, v^	241 (95)^i^	240 (96)^i^	253 (97)^i^	236 (97)^i^	<0.001
Yogurt	1,218 (95)	255 (95)	243 (96)	237 (95)	250 (96)	233 (96)	0.953
All products	1,060 (83)	178 (66)^ii, iii, iv, v^	220 (87)^i^	226 (90)^i^	226 (87)^i^	210 (86)^i^	<0.001
This label makes it easy to understand the amount of sugar in the food product ^a^^,^^b^	843 (67)	133 (51)^ii, iii, iv, v^	199 (80)^i, iii, iv^	164 (66)^i, ii, v^	157 (61)^i, ii, v^	190 (79)^i, iii, iv^	<0.001

*Number superscripts (e.g.,^i, ii, iii^)indicate that a result is significantly different from the study condition with the corresponding number based on Bonferroni-corrected post hoc tests with an alpha set at 0.01*.

a *Proportion of people who agree by the summarising points 5, 6, and 7 from a 7-points Likert scale where 1 = strongly disagree and 7 = strongly agree*.

b *Different sample size for this question (n = 1,257)*.

*NIP, Nutrition Information Panel; FoP, Front-of-Package*.

The odds ratio for the understanding outcome by label condition is presented in Table 3. Participants in all intervention conditions were more likely to identify products with the highest sugar compared to the control condition, although participants who saw the FoP conditions (iii and iv) had the highest odds ratios for that. The octagonal warning (iii) had the best performance for whole-grain biscuits, while the magnifying glass warning (iv) produced more correct answers for the cereal bar and yoghourt categories.

**Table 3:** Odds ratios (OR) and 95% confidence intervals (CI) for primary (understanding) outcome (*n* = 1,277).

**Variables**	**OR (95%CI)**		
	**Whole-grain biscuit**	**Cereal bar**	**Yogurt**
**Label condition**			
i. Control (ref)	–	–	–
ii. Proposed NIP	3.91 (2.24–6.83)^*^	2.85 (1.42–5.71)^*^	1.08 (0.46–2.53)
iii. Proposed NIP + FoP octagonal warning	4.02 (2.28–7.11)^*^	3.47 (1.61–7.31)^*^	0.88 (0.40–2.00)
iv. Proposed NIP + FoP magnifying glass warning	2.92 (1.76–4.86)^*^	5.74 (2.42–13.57)^*^	1.42 (0.58–3.48)
v. Proposed NIP + ‘high in sugar’ text	2.65 (1.59–4.40)^*^	5.26 (2.22–12.47)^*^	1.20 (0.50–2.87)

* *p-value at < 0.01*.

*NIP, Nutrition Information Panel; FoP, Front-of-Package; CI, Confidence Interval*.

When asked whether the label format makes it easier to identify the sugar content of the product, participants in all intervention conditions had higher proportions of agreement than those in the control condition. A significant difference was also found when comparing the intervention conditions on this variable, with participants in the NIP only conditions [(ii) and (v)] found to have a higher proportion of agreement than participants in the NIP plus FoP conditions [(iii) and (iv)] (Table 3). However, this distinction between the intervention conditions was not found in the sensitivity analysis.

### Food Choice (Secondary Outcome)

There were no significant differences in the proportions of participants who chose high-in-sugar products between the label conditions, overall or by food category. The FoP warning conditions had the lowest proportions of participants who chose products high-in-sugar for whole-grain biscuits and yoghourts, but the differences were not significant. Moreover, participants in the FoP octagonal warning condition (iii) had the lowest proportion of participants who chose high-in-sugar products through all three sets of food categories. In contrast, the control (i) and proposed NIP (ii) conditions had the highest proportion of agreement regarding the question of whether the label format influenced participants' choices (Table 4). Although results were not statistically significant, in general, FoP warning seems to be useful to influence consumers to choose products with lower sugar content. In sensitivity analyses, where only participants who recalled seeing the label formats tested during the survey were included, results remained similar (Supplementary Table S1). The proportion of participants who chose the “I would not choose any of these products” option is shown in Table 4. While a low number of participants did not choose any product throughout all food categories (3.9%), higher proportions were found by food category with 32, 31, and 19% for the whole-grain biscuit, cereal bar, and yoghourt categories, respectively.

**Table 4:** Participants' food choices for high-in-sugar products and perceived influence of the label in their choices [n (%)], by study arm (*n* = 1,277).

**Outcomes**	**Total sample** **(*n* = 1,277)**	**i. Control group** **(*n* = 269)**	**ii. Proposed NIP (*n* = 254)**	**iii. Proposed NIP + FoP octagonal warning** **(*n* = 250)**	**iv. Proposed NIP + FoP magnifying glass warning (*n* = 261)**	**v. Proposed NIP + ‘high in sugar’ text** **(*n* = 243)**	***p*-value**
**Food choice**							
The proportion of participants who chose a high-in-sugar option							
Whole-grain biscuits	342 (27)	85 (32)	68 (27)	58 (23)	61 (23)	70 (29)	0.067
Cereal bars	484 (38)	97 (36)	96 (38)	92 (37)	97 (37)	102 (42)	0.568
Yogurts	273 (21)	66 (25)	55 (22)	43 (17)	60 (23)	49 (20)	0.156
All products	97 (8)	23 (9)	19 (8)	13 (5)	21 (8)	21 (9)	0.583
The proportion of participants who chose the 'I wouldn't choose any of these products' option							
Whole-grain biscuits	406 (32)	87 (32)	77 (30)	87 (35)	77 (30)	78 (32)	0.740
Cereal bars	394 (31)	92 (34)	80 (32)	71 (28)	78 (30)	73 (30)	0.670
Yogurts	248 (19)	55 (20)	54 (21)	40 (16)	50 (19)	49 (20)	0.610
All products	50 (4)	9 (3)	13 (5)	10 (4)	9 (3)	9 (4)	0.845
This label has influenced my food choices in this survey^a^^,^^b^	713 (57)	154 (59)^iv^	165 (67)^iii, iv^	124 (50)^ii^	123 (48)^i, ii, v^	147 (61)^iv^	<0.001

*Number superscripts (e.g., ^i, ii, iii^) indicate that a result is significantly different from the study condition with the corresponding number based on Bonferroni-corrected post hoc tests with an alpha set at 0.01*.

a *Proportion of people who agree by the summarising points 5, 6, and 7 from a 7-points Likert scale where 1 = strongly disagree and 7 = strongly agree*.

b *Different sample size for this question (n = 1,257)*.

*NIP, Nutrition Information Panel; FoP, Front-of-Package*.

**Figure 3:**
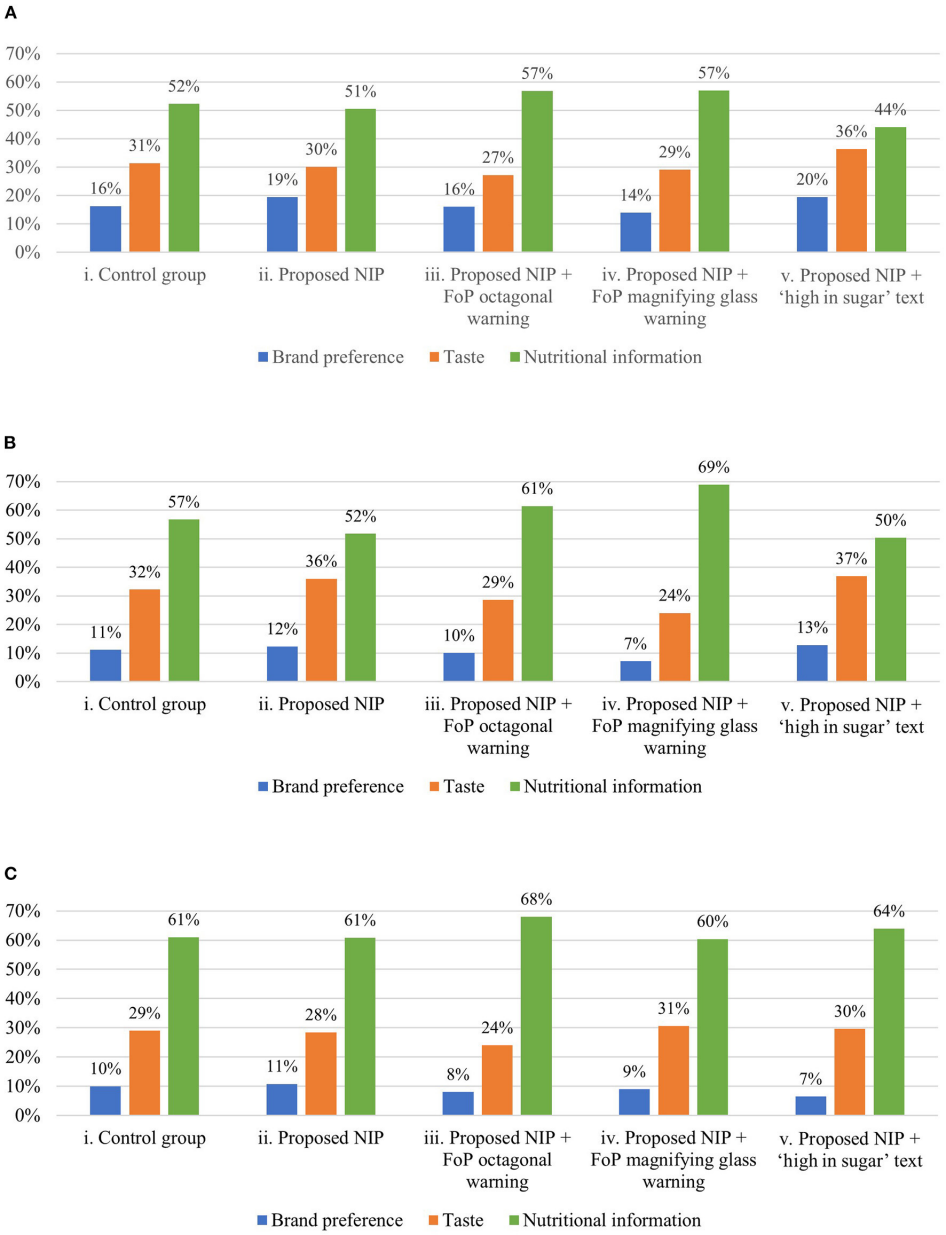
Proportional distribution of each reason for participants' food choices, by food category. **(A)** Reason of product' choice: whole-grain biscuit. **(B)** Reason of product' choice: cereal bar. **(C)** Reason of product' choice: yoghurt. NIP, Nutrition Information Panel. FoP, Front-of-Package.

The most frequent reason for participants' food choices was “Nutrition information” across all three food categories (Figure 3). There were no differences between the reasons for participants' choices across the label conditions for the whole-grain biscuit and yoghourt categories. For the cereal bar category, participants more frequently selected “Nutritional information” in the proposed NIP plus magnifying glass condition (iv) than in the control (i), proposed NIP only (ii), and proposed NIP plus high in sugar text (v) conditions (*p* < 0.05).

## Discussion

Overall, our results showed a clear demand for sugar information to be made available on the labels of packaged foods to inform consumers during food shopping. Compared with the control condition, all the sugar label formats have increased study participants' understanding of the sugar content of the products. However, none of them significantly decreased consumers' choices for products high in sugar.

The findings for understanding from this study are in line with previous research from other countries that have shown that information about sugar on labels increases consumers' understanding of the sugar level of packaged foods (35–39). Participants who saw the FoP warning label conditions were more likely to correctly identify products with the highest content of sugar than participants in the NIP only conditions. These results are aligned with a key aim of FoP nutrition labels being to provide nutrition information in a more understandable way for consumers (16). In contrast to these experimental results, the participants perceived the NIP only conditions as more straightforward to identify the sugar content of the products than when FoP warning labels were also presented. This result may be partially due to the type of understanding task used in this survey, where participants had to identify the product with the highest sugar content. It requires consumers to check the sugar content in grammes in the NIP and then interpret it more than knowing if a product is high-in-sugar or not. A previous study also found that nutrient warnings were perceived as not containing enough information for consumers' needs (40). In terms of consumers' understanding of the nutrition composition of foods, both sources of information (NIP and FoP) can therefore be useful in a complementary way. While the FoP warning labels allow consumers to correctly, quickly, and easily identify products containing excessive amounts of critical nutrients (41, 42), the NIP provides them with specific nutrient amounts to permit more detailed product comparisons.

Our results on consumer understanding identified differences according to food category. While participants' understanding was found to increase significantly for whole-grain biscuits and cereal bars, this was not the case for the yoghourt category. Participants in all study arms had high proportions of correct answers when asked to identify which yoghourt was the highest in sugar (above 94%). Some explanations could be attributed to this. First, yoghourt has been previously described in other populations as one of the products that consumers are more likely to read nutrition information for when shopping (43). Second, because all the carbohydrate content of yoghourts are sugars (naturally present as lactose or added from other sources), participants in the control group may have used the carbohydrate information available in the NIP as a guide to sugar content. This interpretation is reinforced by the fact that during our focus groups, participants incorrectly associated all the carbohydrate content of packaged food as equal to the sugar content. For the whole-grain biscuit and cereal bar categories, other sources of carbohydrates, such as flour or nuts, were present in the products' composition, and the carbohydrate content in the NIP by itself was not enough to help consumers identify the sugar content of these products.

Many studies have tested the influence of different label formats on consumers' food choices or purchases, and mixed results have been found according to the types of label and food category tested, the methodology used, and participants' nationality (37, 39, 44–50). A systematic review investigating sugar label formats and their influence on consumers' food choice has demonstrated that interpretive information (e.g., colours, “high in sugar” text, warnings, or health messages) is more effective than the standard NIP in encouraging consumers to choose foods with less sugar (31). The results of the present study showed that the sugar label formats tested did not significantly decrease consumers' choices for products high in sugar. Nevertheless, the proposed NIP plus an octagonal FoP label (iii) had the lowest proportion of participants choosing high-in-sugar products for whole-grain biscuits, yoghourts, and across all products. Studies conducted in neighbouring Latin American countries have demonstrated that FoP octagonal warning labels effectively reduce consumer choice of foods high in critical nutrients (47, 49, 51, 52).

The non-significant effects observed for food choice in the present study could be related to our sample's sociodemographic and behavioural profile. Most of the participants in our study were female and had a high educational level, and these factors have been previously described as influencing the use and understanding of nutritional information on labels (53, 54). In fact, 84% of the participants said they used nutritional information frequently during their routine shopping, which would explain the high proportion of participants who selected nutritional quality as the main reason for their choices during the survey. It is known that the use of nutritional information is associated with the level of understanding of this information by consumers (43, 54, 55), which can lead to healthier food choices (56, 57). In our results, a high proportion of participants (83%) correctly understood the sugar content of all products tested, which would help to explain the low proportion of participants who choose high-in-sugar products and the non-significant difference between the label conditions.

### Study Limitations and Strengths

Strengths of our study include the inclusion of a qualitative phase to support the development of the subsequent survey, the voluntary participation of a large number of Brazilian consumers from various sociodemographic backgrounds, the investigation of two outcomes related to food label use (understanding and food choice), and the comparison across multiple types of sugar label formats using a randomised approach. In addition, a potential learning bias was minimised by testing the food choice task first and then objective understanding, as well as using randomisation of the presentation order across food categories. While product brand is understood to be a key aspect driving consumers' food choices (58), the impact of brand preference seemed to be minimal in our study. By asking participants why they had chosen specific food products, we were able to assess the impact of using real brands in this experiment. The results indicate that brand preference was the least frequent reason for their choices, with no differences found across the conditions. This may have been due to the fact that we had a high-educated and health-concerned sample of participants who give more importance to the nutritional aspects of food products rather than the products' brand.

Some limitations need to be acknowledged. The choice for young food label users for the focus groups was made to explore perceptions of an audience with lived experience on the topic of investigation (food label usage) who commonly have a high sugar intake. However, participant perspectives in the qualitative phase can be different from people with other socioeconomic characteristics, mainly because our sample was highly educated. In this sense, findings from the qualitative phase have limited generalisation. Similarly, although the online survey was shared with several groups of people, our sample has a sociodemographic profile different from the general adult population in Brazil (59), requiring caution in the extrapolation of results. It is also important to note that certain elements of the study design are likely to have influenced the results. First, the study was conducted through an online platform, which was the only feasible way to get data collected due to the COVID-19 restrictions enforced in Brazil when the data was collected. Because we collected the data via an online platform that did not allow an interactive visualisation of the products, we only displayed the products' image of the front panel of the product. Because of this, the FoP formats were embedded in the images while the NIPs were displayed on the side. This arrangement gave FoP and back of pack (NIP + list of ingredients) information the same weight because participants saw them simultaneously, which is different from what they see for real-world packaged products. This may have driven participants to pay more attention to the NIP than the FoP, as we found in our results. However, a previous study has also reported a lower proportion of participants recalling seeing FoP black-and-white warning symbol during food choice tasks (45). We tried to minimise this effect by instructing participants to zoom in on the images of the products during the tasks, but we were unable to measure if they had done so. Nevertheless, caution should be taken when using our findings to inform public policies. Another point is that many participants selected the “I wouldn't choose any of these products” option during the food choice task, reducing the sample size for this outcome and reducing the statistical power to find significant differences between the groups.

It is also worth noting that only three food categories were tested, limiting the magnitude of the effects compared to studies measuring the overall shopping cart or in a real-world environment. However, in our case, the number of sets and products within the sets had to remain limited given that two outcomes were investigated in the same survey, and the questionnaire could not be too long for participants to complete. Finally, because the study was conducted before the approved changes in the food label rules in Brazil, we could not test the exact FoP format that will be implemented soon. However, our label condition (iv) [FoP magnifying glass warning] is similar to the approved format in Brazil, which allows for some inference of the effects found in our study to the approved format.

### Practical Implications and Future Research

The results support the new changes in Brazil's label policy, requiring a mandatory declaration of the total and added sugar contents in grammes displayed in the nutrition information panel of all packaged foods available for sale in Brazil by October 2022. These changes will help consumers easily and quickly identify the sugar content of packaged foods during their shopping, allowing comparisons between products within the same food category. Moreover, although results were non-significant, participants who had seen the FoP conditions had the lowest proportion choosing high-in-sugar products, which suggests that the inclusion of a mandatory FoP is beneficial. Our evidence suggests that an octagonal front-of-pack warning similar to the one used in other Latin-American countries would have the best impact on incentivising Brazilian consumers to reduce their choices for products high in sugar. This is relevant considering recent evidence showing that most packaged foods and beverages sold in Brazil have added sugar ingredients in their composition (60), and that the Brazilian population is eating more added sugar than recommended by the WHO (11).

Furthermore, the enforcement of any FoP on labels should be complemented by government campaigns that educate consumers on how to use the labels and the differences between sugars naturally found in fruit, vegetables and dairy products and sugars added to the packaged food products, as well as the differences between sugars and carbohydrates. In addition, as found during the focus groups, participants seem to trust information endorsed by the Health Ministry more than any disclosure made by the food industry. Future researchers should use the newly approved FoP formats being implemented in Brazil to test consumers' perceptions, understanding, and food choices in larger samples. In addition, groups other than university students should be targeted for future qualitative research on food label perceptions to ensure representation of a wide range of views and experiences. We also suggest that real-world supermarkets studies be conducted to investigate the effects of sugar label formats during real decision-making processes.

## Conclusions

Information about the sugar content of packaged foods displayed on either the NIP or FoP labels is a meaningful strategy to help Brazilian consumers compare products and correctly identify foods with higher sugar content among products within the same food category. While no significant difference across labels was observed for food choices, the sugar content displayed in the NIP plus an octagonal warning demonstrated the highest performance in stimulating consumers to avoid high-in-sugar products. Additional research is needed to understand how sugar label formats impact the understanding and food choices of Brazilian samples from different socioeconomic groups. Policymakers and researchers should be encouraged to investigate the efficacy of the approved food label changes in Brazil on consumer behaviour.

## Data Availability Statement

The raw data supporting the conclusions of this article will be made available by the authors, without undue reservation.

## Ethics Statement

The studies involving human participants were reviewed and approved by the Human Research Ethics Committee (CEPSH) of the University of Santa Catarina (Process No. 3063750). The patients/participants provided their written informed consent to participate in this study.

## Author Contributions

TS was responsible for conceptualisation, methodology, formal analysis, investigation, writing—original draft, and writing—review and editing. AF and RP were responsible for conceptualisation, methodology, writing—review and editing, and supervision. MS was responsible for formal analysis and writing—review and editing. SP was responsible for conceptualisation, methodology, resources, writing—review and editing, and supervision. NK, GB, and PU were responsible for conceptualisation and writing—review and editing. All authors contributed to the article and approved the submitted version.

## Funding

This study was financed in part by the Brazilian Federal Agency for Support and Evaluation of Graduate Education (CAPES) in the form of a scholarship awarded to TS in Brazil and during her internship carried out at the George Institute for Global Health, Sydney, Australia (Award Code No. 41/2018). The authors thank the Brazilian National Council for Scientific and Technological Development (CNPq) of the Ministry of Science, Technology, Innovation, and Communication for funding the wider project Nutrition Labelling of Brazilian Foods: A Thematic Analysis of the Use of Food Labels and their Influence on Consumers' Choices (Grant No. 440040/2014-0) and for the financial support in the form of a research productivity scholarship granted to RP (Award No. 305068/2018-0). None of the sponsors influenced the study design, data collection or analysis, manuscript preparation or revision, or publication decisions.

## Conflict of Interest

The authors declare that the research was conducted in the absence of any commercial or financial relationships that could be construed as a potential conflict of interest.

## Publisher's Note

All claims expressed in this article are solely those of the authors and do not necessarily represent those of their affiliated organizations, or those of the publisher, the editors and the reviewers. Any product that may be evaluated in this article, or claim that may be made by its manufacturer, is not guaranteed or endorsed by the publisher.
