# Supplementary material for: Consumers' Response to Sugar Label Formats in Packaged Foods: A Multi-Methods Study in Brazil
Source: Front Nutr. 2022 Jun 16;9:896784. doi: 10.3389/fnut.2022.896784 (PMC9245067; doi:10.3389/fnut.2022.896784)
Supplement: Supplementary file 1 [file Data_Sheet_1.pdf]

Supplementary material

Figure S1. Stimuli used in the online survey (whole-grain biscuit example).

i. Control condition

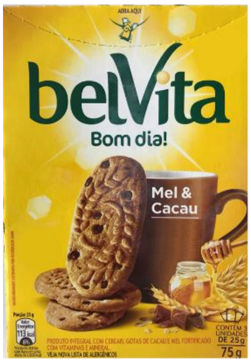

Porção: 30g	Medida caseira: 3 unidades	
	Qnt. por porção	%DV
Energia	135 kcal	6,8
Carboidratos	20,4 g	6,8
Proteínas	2,3 g	3,0
Gorduras totais	4,8 g	8,7
Gorduras saturadas	0,7 g	3,3
Gorduras trans	0 g	
Fibras	1,3 g	5,3
Sódio	63,6 mg	2,7

Lista de ingredientes: cereais [farinha de trigo enriquecida com ferro, ácido fólico e vitaminas B3, B2 e B1, cereais integrais (farinha de trigo integral, aveia em flocos, farinha de cevada e farinha de centeio)], açúcar, óleo vegetal, mel, gotas de cacau, açúcar invertido, carbonato de cálcio, sal, leite em pó desnatado, vitaminas: vitamina D e vitamina E, fermentos químicos: bicarbonato de sódio, bicarbonato de amônio e fosfato monocálcico, aromatizantes e emulsificantes: lectitina de soja e ésteres de ácido diacetil tartárico e mono e dilgicerídeos.

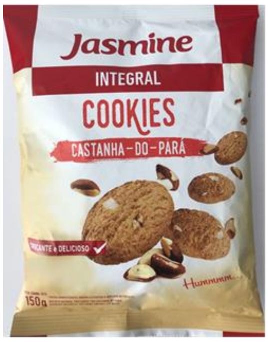

Porção: 30g	Medida caseira: 6 unidades	
	Qnt. por porção	%DV
Energia	127 kcal	6,4
Carboidratos	20,0 g	6,7
Proteínas	2,5 g	3,3
Gorduras totais	4,1 g	7,5
Gorduras saturadas	0,9 g	4,1
Gorduras trans	0 g	
Fibras	2,5 g	10,0
Sódio	40,0 mg	1,7

Lista de ingredientes: Farinha de trigo integral, farinha de trigo enriquecida com ferro e ácido fólico, açúcar mascavo, melado de cana, óleos vegetais de milho e ou girassol e ou algodão), castanha-do-Pará, amido, maltodextrina, estabilizante natural maltitol, fermentos fosfato monocálcico, bicasrbonato de sódio e bicarbonato de amônio, emulsificante natural lecitina de soja e aromas.

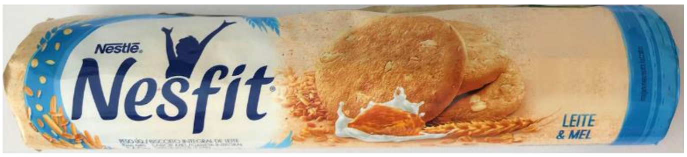

Porção: 30g	Medida caseira: 6 unidades	
	Qnt. por porção	%DV
Energia	127 kcal	6,4
Carboidratos	19,0 g	6,3
Proteínas	2,6 g	3,5
Gorduras totais	4,6 g	8,4
Gorduras saturadas	0,6 g	2,7
Gorduras trans	0 g	
Fibras	2,7 g	10,8
Sódio	98,0 mg	4,1

Lista de ingredientes: cereais integrais (farinha de trigo, aveia em flocos, quinoa e farinha de centeio integral), açúcar, óleo vegetal, amido, fibra de trigo, açúcar invertido, leite em pó integral, soro de leite, sal, fermentos químicos (bicarbonato de amônio, bicarbonato de sódio e fosfato monocálcico), aromatizantes, emulsificante (lecitina de soja) e antioxidante (TBHQ).

ii. Proposed NIP

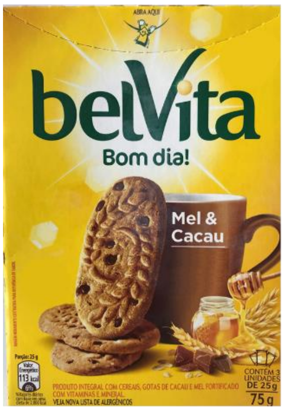

Porção: 30g	Medida caseira: 3 unidades	
	Qnt. por porção	%DV
Energia	135 kcal	6,8
Carboidratos	20,4 g	6,8
Açúcares totais	7,7 g	-
Açúcares de adição	7,7 g	-
Proteínas	2,3 g	3,0
Gorduras totais	4,8 g	8,7
Gorduras saturadas	0,7 g	3,3
Gorduras trans	0 g	-
Fibras	1,3 g	5,3
Sódio	63,6 mg	2,7

Lista de ingredientes: cereais [farinha de trigo enriquecida com ferro, ácido fólico e vitaminas B3, B2 e B1, cereais integrais (farinha de trigo integral, aveia em flocos, farinha de cevada e farinha de centeio)], açúcar, óleo vegetal, mel, gotas de cacau, açúcar invertido, carbonato de cálcio, sal, leite em pó desnatado, vitaminas: vitamina D e vitamina E, fermentos químicos: bicarbonato de sódio, bicarbonato de amônio e fosfato monocálcico, aromatizantes e emulsificantes: lecitina de soja e ésteres de ácido diacetil tartárico e mono e dilgicerídeos.

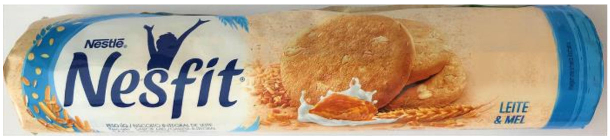

Porção: 30g	Medida caseira: 6 unidades	
	Qnt. por porção	%DV
Energia	127 kcal	6,4
Carboidratos	19,0 g	6,3
Açúcares totais	5,7 g	-
Açúcares de adição	5,7 g	-
Proteínas	2,6 g	3,5
Gorduras totais	4,6 g	8,4
Gorduras saturadas	0,6 g	2,7
Gorduras trans	0 g	-
Fibras	2,7 g	10,8
Sódio	98,0 mg	4,1

Lista de ingredientes: cereais integrais (farinha de trigo, aveia em flocos, quinoa e farinha de centeio integral), açúcar, óleo vegetal, amido, fibra de trigo, açúcar invertido, leite em pó integral, soro de leite, sal, fermentos químicos (bicarbonato de amônio, bicarbonato de sódio e fosfato monocálcico), aromatizantes, emulsificante (lecitina de soja) e antioxidante (TBHQ).

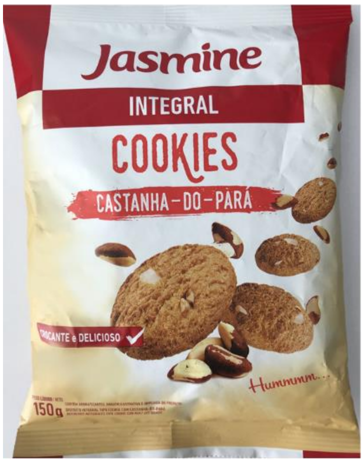

Porção: 30g	Medida caseira: 6 unidades	
	Qnt. por porção	%DV
Energia	127 kcal	6,4
Carboidratos	20,0 g	6,7
Açúcares totais	1,2 g	-
Açúcares de adição	1,2 g	-
Proteínas	2,5 g	3,3
Gorduras totais	4,1 g	7,5
Gorduras saturadas	0,9 g	4,1
Gorduras trans	0 g	-
Fibras	2,5 g	10,0
Sódio	40,0 mg	1,7

Lista de ingredientes: Farinha de trigo integral, farinha de trigo enriquecida com ferro e ácido fólico, melado de cana, óleos vegetais de milho e ou girassol e ou algodão), castanha-do-Pará, amido, maltodextrina, estabilizante natural maltitol, fermentos fosfato monocálcico, bicasrbonato de sódio e bicarbonato de amônio, emulsificante natural lecitina de soja e aromas.

iii. Proposed NIP + FoP octagonal warning

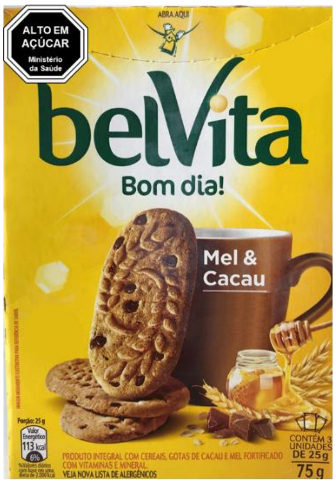

Porção: 30g	Medida caseira: 3 unidades	
	Qnt. por porção	%DV
Energia	135 kcal	6,8
Carboidratos	20,4 g	6,8
Açúcares totais	7,7 g	-
Açúcares de adição	7,7 g	-
Proteínas	2,3 g	3,0
Gorduras totais	4,8 g	8,7
Gorduras saturadas	0,7 g	3,3
Gorduras trans	0 g	-
Fibras	1,3 g	5,3
Sódio	63,6 mg	2,7

Lista de ingredientes: cereais [farinha de trigo enriquecida com ferro, ácido fólico e vitaminas B3, B2 e B1, cereais integrais (farinha de trigo integral, aveia em flocos, farinha de cevada e farinha de centeio)], açúcar, óleo vegetal, mel, gotas de cacau, açúcar invertido, carbonato de cálcio, sal, leite em pó desnatado, vitaminas: vitamina D e vitamina E, fermentos químicos: bicarbonato de sódio, bicarbonato de amônio e fosfato monocálcico, aromatizantes e emulsificantes: lectitina de soja e ésteres de ácido diacetil tartárico e mono e dilgicerídeos.

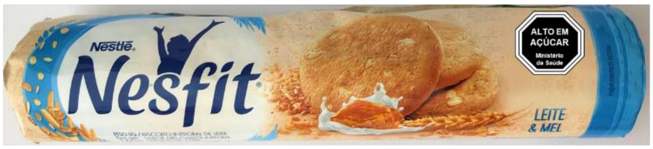

Porção: 30g	Medida caseira: 6 unidades	
	Qnt. por porção	%DV
Energia	127 kcal	6,4
Carboidratos	19,0 g	6,3
Açúcares totais	5,7 g	-
Açúcares de adição	5,7 g	-
Proteínas	2,6 g	3,5
Gorduras totais	4,6 g	8,4
Gorduras saturadas	0,6 g	2,7
Gorduras trans	0 g	-
Fibras	2,7 g	10,8
Sódio	98,0 mg	4,1

Lista de ingredientes: cereais integrais (farinha de trigo, aveia em flocos, quinoa e farinha de centeio integral), açúcar, óleo vegetal, amido, fibra de trigo, açúcar invertido, leite em pó integral, soro de leite, sal, fermentos químicos (bicarbonato de amônio, bicarbonato de sódio e fosfato monocálcico), aromatizantes, emulsificante (lecitina de soja) e antioxidante (TBHQ).

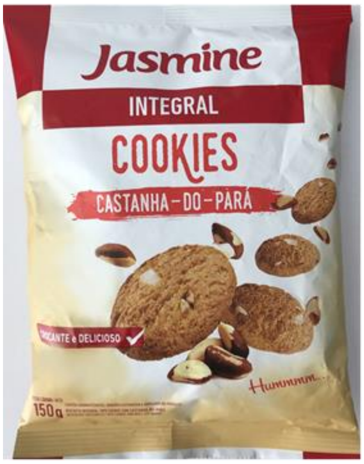

Porção: 30g	Medida caseira: 6 unidades	
	Qnt. por porção	%DV
Energia	127 kcal	6,4
Carboidratos	20,0 g	6,7
Açúcares totais	1,2 g	-
Açúcares de adição	1,2 g	-
Proteínas	2,5 g	3,3
Gorduras totais	4,1 g	7,5
Gorduras saturadas	0,9 g	4,1
Gorduras trans	0 g	-
Fibras	2,5 g	10,0
Sódio	40,0 mg	1,7

Lista de ingredientes: Farinha de trigo integral, farinha de trigo enriquecida com ferro e ácido fólico, melado de cana, óleos vegetais de milho e ou girassol e ou algodão), castanha-do-Pará, amido, maltodextrina, estabilizante natural maltitol, fermentos fosfato monocálcico, bicasrbonato de sódio e bicarbonato de amônio, emulsificante natural lecitina de soja e aromas.

iv. Proposed NIP + FoP magnifying glass warning

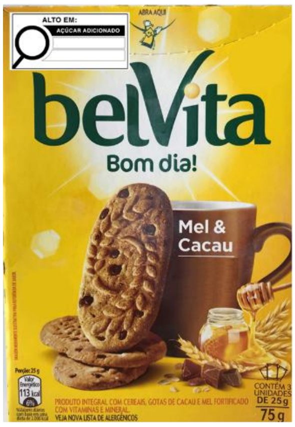

Porção: 30g	Medida caseira: 3 unidades	
	Qnt. por porção	%DV
Energia	135 kcal	6,8
Carboidratos	20,4 g	6,8
Açúcares totais	7,7 g	-
Açúcares de adição	7,7 g	-
Proteínas	2,3 g	3,0
Gorduras totais	4,8 g	8,7
Gorduras saturadas	0,7 g	3,3
Gorduras trans	0 g	-
Fibras	1,3 g	5,3
Sódio	63,6 mg	2,7

Lista de ingredientes: cereais [farinha de trigo enriquecida com ferro, ácido fólico e vitaminas B3, B2 e B1, cereais integrais (farinha de trigo integral, aveia em flocos, farinha de cevada e farinha de centeio)], açúcar, óleo vegetal, mel, gotas de cacau, açúcar invertido, carbonato de cálcio, sal, leite em pó desnatado, vitaminas: vitamina D e vitamina E, fermentos químicos: bicarbonato de sódio, bicarbonato de amônio e fosfato monocálcico, aromatizantes e emulsificantes: lectitina de soja e ésteres de ácido diacetil tartárico e mono e dilgicerídeos.

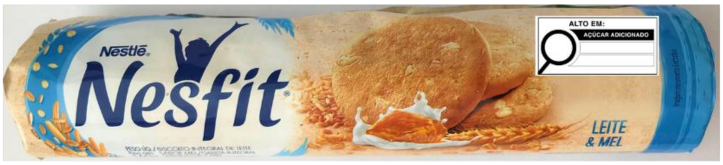

Porção: 30g	Medida caseira: 6 unidades	
	Qnt. por porção	%DV
Energia	127 kcal	6,4
Carboidratos	19,0 g	6,3
Açúcares totais	5,7 g	-
Açúcares de adição	5,7 g	-
Proteínas	2,6 g	3,5
Gorduras totais	4,6 g	8,4
Gorduras saturadas	0,6 g	2,7
Gorduras trans	0 g	-
Fibras	2,7 g	10,8
Sódio	98,0 mg	4,1

Lista de ingredientes: cereais integrais (farinha de trigo, aveia em flocos, quinoa e farinha de centeio integral), açúcar, óleo vegetal, amido, fibra de trigo, açúcar invertido, leite em pó integral, soro de leite, sal, fermentos químicos (bicarbonato de amônio, bicarbonato de sódio e fosfato monocálcico), aromatizantes, emulsificante (lecitina de soja) e antioxidante (TBHQ).

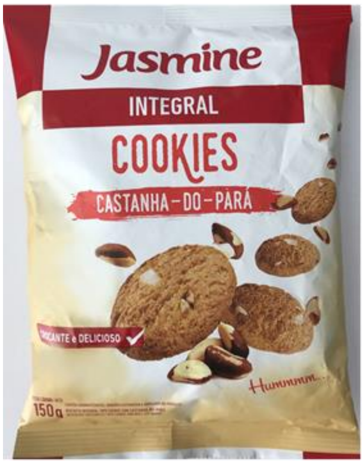

Porção: 30g	Medida caseira: 6 unidades	
	Qnt. por porção	%DV
Energia	127 kcal	6,4
Carboidratos	20,0 g	6,7
Açúcares totais	1,2 g	-
Açúcares de adição	1,2 g	-
Proteínas	2,5 g	3,3
Gorduras totais	4,1 g	7,5
Gorduras saturadas	0,9 g	4,1
Gorduras trans	0 g	-
Fibras	2,5 g	10,0
Sódio	40,0 mg	1,7

Lista de ingredientes: Farinha de trigo integral, farinha de trigo enriquecida com ferro e ácido fólico, melado de cana, óleos vegetais de milho e ou girassol e ou algodão), castanha-do-Pará, amido, maltodextrina, estabilizante natural maltitol, fermentos fosfato monocálcico, bicasrbonato de sódio e bicarbonato de amônio, emulsificante natural lecitina de soja e aromas.

v. Proposed NIP + 'high in sugar' text

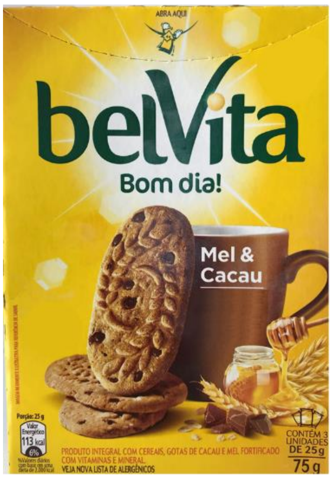

Porção: 30g	Medida caseira: 3 unidades	
	Qnt. por porção	%DV
Energia	135 kcal	6,8
Carboidratos	20,4 g	6,8
Açúcares totais	7,7 g	-
Açúcares de adição	7,7 g **(alto em)**	-
Proteínas	2,3 g	3,0
Gorduras totais	4,8 g	8,7
Gorduras saturadas	0,7 g	3,3
Gorduras trans	0 g	-
Fibras	1,3 g	5,3
Sódio	63,6 mg	2,7

Lista de ingredientes: cereais [farinha de trigo enriquecida com ferro, ácido fólico e vitaminas B3, B2 e B1, cereais integrais (farinha de trigo integral, aveia em flocos, farinha de cevada e farinha de centeio)], açúcar, óleo vegetal, mel, gotas de cacau, açúcar invertido, carbonato de cálcio, sal, leite em pó desnatado, vitaminas: vitamina D e vitamina E, fermentos químicos: bicarbonato de sódio, bicarbonato de amônio e fosfato monocálcico, aromatizantes e emulsificantes: lectitina de soja e ésteres de ácido diacetil tartárico e mono e dilgicerídeos.

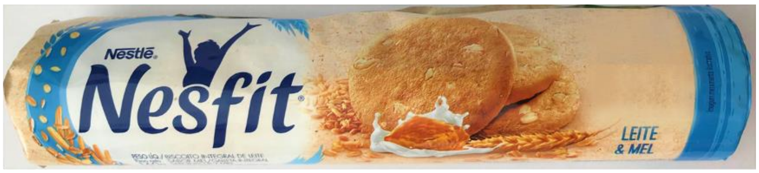

Porção: 30g	Medida caseira: 6 unidades	
	Qnt. por porção	%DV
Energia	127 kcal	6,4
Carboidratos	19,0 g	6,3
Açúcares totais	5,7 g	-
Açúcares de adição	5,7 g **(alto em)**	-
Proteínas	2,6 g	3,5
Gorduras totais	4,6 g	8,4
Gorduras saturadas	0,6 g	2,7
Gorduras trans	0 g	-
Fibras	2,7 g	10,8
Sódio	98,0 mg	4,1

Lista de ingredientes: cereais integrais (farinha de trigo, aveia em flocos, quinoa e farinha de centeio integral), açúcar, óleo vegetal, amido, fibra de trigo, açúcar invertido, leite em pó integral, soro de leite, sal, fermentos químicos (bicarbonato de amônio, bicarbonato de sódio e fosfato monocálcico), aromatizantes, emulsificante (lecitina de soja) e antioxidante (TBHQ).

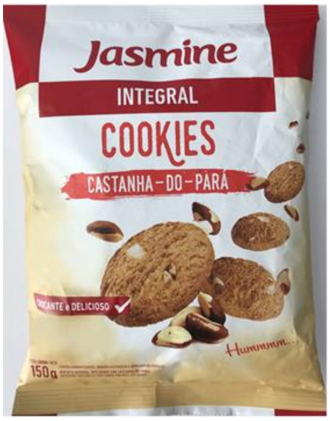

Porção: 30g	Medida caseira: 6 unidades	
	Qnt. por porção	%DV
Energia	127 kcal	6,4
Carboidratos	20,0 g	6,7
Açúcares totais	1,2 g	-
Açúcares de adição	1,2 g	-
Proteínas	2,5 g	3,3
Gorduras totais	4,1 g	7,5
Gorduras saturadas	0,9 g	4,1
Gorduras trans	0 g	-
Fibras	2,5 g	10,0
Sódio	40,0 mg	1,7

Lista de ingredientes: Farinha de trigo integral, farinha de trigo enriquecida com ferro e ácido fólico, melado de cana, óleos vegetais de milho e ou girassol e ou algodão), castanha-do-Pará, amido, maltodextrina, estabilizante natural maltitol, fermentos fosfato monocálcico, bicasrbonato de sódio e bicarbonato de amônio, emulsificante natural lecitina de soja e aromas.

Table S1. Analyses of understanding of sugar level and choice of high-in-sugar products (n, %) among participants who reported noticing the label during the survey, by food category and label condition (n = 992).

Outcomes	i. Control group (n = 227)	ii. Proposed NIP (n = 218)	iii. Proposed NIP + FoP octagonal warning (n = 166)	iv. Proposed NIP + FoP magnifying glass warning (n = 166)	v. Proposed NIP + 'high in sugar' text (n = 215)	p-value
**Understanding**						
Proportion of correct answers about which product had the highest sugar content						
Whole-grain biscuits	175 (77)[ii, iii, iv, v]	205 (94)[i]	153 (92)[i]	151 (91)[i]	196 (91)[i]	<0.001
Cereal bars	201 (89)[iv, v]	206 (95)	156 (94)	160 (96)[i]	209 (97)[i]	<0.002
Yogurt	215 (95)	209 (96)	154 (93)	159 (96)	208 (97)	0.440
All products	153 (67)[ii, iii, iv, v]	191 (88)[i]	147 (89)[i]	147 (89)[i]	191 (89)[i]	<0.001
'This label makes it easy to understand the amount of sugar in the food product'[1,2]	120 (53)[ii, iii, iv, v]	183 (84)[i]	141 (85)[i]	131 (79)[i]	177 (82)[i]	<0.001
**Food choice**						
Proportion of participants who chose a high-in-sugar option						
Whole-grain biscuits	73 (32)	55 (25)	34 (21)	35 (21)	58 (27)	0.071
Cereal bars	80 (35)	78 (36)	57 (34)	55 (31)	87 (41)	0.536
Yogurts	51 (22)	44 (20)	22 (13)	39 (24)	39 (18)	0.078
All products	21 (9)	16 (7)	4 (2)	13 (8)	17 (8)	0.115
'This label has influenced my food choices in this survey[1,2]	143 (63)	152 (70)	105 (63)	107 (65)	136 (63)	0.549

Number superscripts (e.g., [i, ii, iii]) indicate that a result is significantly different from the study condition with the corresponding number based on Bonferroni-corrected post hoc tests with alpha set at 0.01. [1]Proportion of people who agree by the summarising points 5, 6, and 7 from a 7-points Likert scale where 1= strongly disagree and 7= strongly agree. [2]Different sample size for this question (*n* = 1,257). Abbreviation: NIP, Nutrition Information Panel; FoP, Front-of-Package.
